# The Effect of Low-Dose Aspirin on Frailty in Older Adults in the Aspirin in Reducing Events in the Elderly Study

**DOI:** 10.1093/geroni/igab046.3009

**Published:** 2021-12-17

**Authors:** Sara Espinoza, A R M Saifuddin Ekram, Robyn Woods, Michael Ernst, Galina Polekhina, John McNeil, Anne Murray, Joanne Ryan

**Affiliations:** 1 University of Texas Health Science Center San Antonio, San Antonio, Texas, United States; 2 Monash University, Monash University, Melbourne, Victoria, Australia; 3 Monash University, Melbourne, Victoria, Australia; 4 University of Iowa, Iowa City, Iowa, United States; 5 Hennepin HealthCare Research Institute, Hennepin HealthCare, Minneapolis, Minnesota, United States

## Abstract

There are no widely accepted pharmacologic treatments for frailty prevention. Since frailty is associated with inflammation, aspirin has the potential to reduce frailty. We investigated whether low-dose aspirin reduces incident frailty in participants of the ASPirin in Reducing Events in the Elderly (ASPREE) trial. In the U.S and Australia, 19,114 healthy community-dwelling individuals aged ≥70 years (U.S. minorities ≥65 years) were enrolled in ASPREE, a double-blind, placebo-controlled trial of 100mg daily low-dose aspirin vs. placebo. Frailty was defined according to a modified Fried frailty definition, and a frailty index which used a deficit accumulation model. Competing risk Cox proportional hazards models were used to compare time to incident frailty for aspirin vs. placebo. At baseline, 2.2% and 8.1% met criteria for frailty by Fried and frailty index criteria, respectively. Over a median of 4.7 years of follow-up, 2252 participants developed incident frailty according to Fried classification, and 4376 according to the frailty deficit accumulation index. There was no difference in the risk of incident frailty between individuals randomized to aspirin versus placebo according to either criteria (Fried frailty HR: 1.03, 95% CI 0.97-1.09, p=0.41; frailty index HR: 1.03, 95% CI 0.97-1.10, p=0.29). Change in frailty over time was not different between the aspirin and placebo treatment arms. The results were consistent across a series of sub-groups, including baseline frailty status. Based on these results, aspirin use in healthy older adults does not reduce incident frailty.

